# Telesurgery: Past, Present, and Future

**DOI:** 10.7759/cureus.2716

**Published:** 2018-05-31

**Authors:** Paul J Choi, Rod J Oskouian, R. Shane Tubbs

**Affiliations:** 1 Surgery, Seattle Science Foundation, Seattle, USA; 2 Neurosurgery, Swedish Neuroscience Institute, Seattle, USA; 3 Neurosurgery, Seattle Science Foundation, Seattle, USA

**Keywords:** telesurgery, virtual interactive presence, travel medicine, telemedicine, robotic surgery, haptic feedback, latency time

## Abstract

Telesurgery uses wireless networking and robotic technology to allow surgeons to operate on patients who are distantly located. This technology not only benefits today’s shortage of surgeons, but it also eliminates geographical barriers that prevent timely and high-quality surgical intervention, financial burden, complications, and often risky long-distance travel. The system also provides improved surgical accuracy and ensures the safety of surgeons. In this paper, we describe the current trend of telesurgery’s innovative developments and its future.

## Introduction and background

Telesurgery is an emerging surgical system that utilizes wireless networking and robotic technology to connect surgeons and patients who are distantly located from one another [1]. The system overcomes today’s shortage of surgeons, geographical inaccessibility of immediate and high-quality surgical care, significant financial burden, potential complications, and long-distance travel [2-5]. This technology not only benefits the patients but also provides technical accuracy and ensures the safety of surgeons.

## Review

Benefits of telesurgery 

Telesurgery provides safe and accurate surgical procedures for patients who are unable to travel a long-distance. With the advancement of robotics and wireless communication technology, this form of surgery is becoming more feasible. Some of the benefits a telesurgery provides are summarized in Table 1.

**Table 1:** A summary of the benefits of telesurgery

Benefits of Telesurgery
Provides high-quality surgery to medically underserved locations [6]such as rural areas, battlefields, and spacecraft [5-8]
Eliminates the need for long-distance travels, along with travel-related financial burden and dangers [2-5,7]
Today’s 3-Dimensional display system provides a shared, high-definition visual feedback to surgeons at different centers simultaneously [5,9]
Allows for surgical collaboration amongst surgeons at different medical centers in real-time [3,10]
Operator’s physiologic tremor can be canceled out in real-time with accelerometer technology [3,11-13], improving surgical accuracy and reducing damage to adjacent healthy tissues [3,12]
Minimized damage to healthy tissues quickens patient recovery [3]

Advancements in telesurgery since 2001

There have been numerous technological advancements in telesurgery since the world’s first telesurgery in 2001, which was conducted by a surgical team in New York, USA using the ZEUS robotic system (Intuitive Surgical, Sunnyvale, CA, USA) [3,7-8]. This project produced a successful two-hour-long laparoscopic cholecystectomy [3,7-8], which was performed on a female patient at a hospital in Strasbourg, France [1,6-7]. The patient had an uneventful recovery [3,7-8].

Innovations in the visual feedback system

In 2014, Shenai et al. introduced Virtual Interactive Presence (VIP), a novel technology that allows remote neurosurgeons to collaborate with a shared 3-Dimensional (3D) display via high-definition binoculars [5,10]. This real-time visual system allows the surgeons to view a merged surgical field display of each other’s hand motions [5]. Shenai et al. carried out successful pterional and suboccipital craniotomies and microscopic approaches to the pineal gland on a cadaveric model using VIP [5,10]. Therefore, VIP would not only be applicable in surgical patient care but would also be useful in surgical training since it allows for a profound real-time interaction among surgeons at different medical centers from around the world [3,10]. However, VIP has a drawback of having a mean latency-time of 760 ± 606 milliseconds, thus further optimization is required [5].

Studying the latency time 

A major problem with telesurgery is latency time, which is defined as the time delay in transferring auditory, visual, and even tactile feedback between the two distant locations [1]. Increased latency time is mainly attributable to network routing problem and congestion, and server overload. The time delay not only generates a lengthy operation but also produces significant surgical inaccuracy [7-8,14], which can risk the safety and delay the recovery of the patient [1,3,7].

According to Wirz et al., an ideal latency time is less than 100 milliseconds and a latency time that is greater than 300 milliseconds produces major inaccuracies in instrument handling [15]. In fact, the first telesurgical cholecystectomy conducted in 2001 had a latency time of 155 milliseconds [7]. Further, a Da Vinci (Intuitive Surgical, Sunnyvale, CA, USA) prototype was used by Nguan et al. in 2008 to check its feasibility in a telesurgical application [14]. However, the prototype produced 340 milliseconds of latency time [14]. Moreover, National Aeronautics and Space Administration (NASA) has conducted experiments in the areas of televascular surgery such as laparoscopic cholecystectomy and abdominal surgery for astronauts [1]. However, a significant time latency is also an issue here [1]. Korte et al. concluded that even the most experienced teleoperator cannot perform with acceptable accuracy and efficiency when the latency time is greater than two seconds, further emphasizing the significance of this variable in telesurgery [7].

Although a latency time of less than 100 milliseconds can be achieved with today’s high-speed fiber optic cables and a dedicated asynchronous transfer mode (ATM), 40 technicians must be present during the surgery to maintain this speed [1,5,8,16]. Interestingly, Xu et al. studied the effects of latency time training and claimed that a degree of inaccuracy from a time delay can be overcome via training the teleoperator [8].

Introduction of haptic feedback technology

The conventional telesurgery system had a major drawback in that it failed to provide tactile information and the operator solely relied on a visual feedback [17]. The technology that enables transmission of tactile information to the teleoperator is termed "haptic feedback." This is a crucial aspect of a wireless robotic surgical system, which enables the operator to feel the consistency of the tissue and the tension within the sutures, prevents damage to the fragile tissues or tearing of the sutures during the operation, and improves the operator’s confidence during surgery [12,17-18].

The first telesurgery prototype that implemented haptic feedback technology was Telelap Alf-x (SOFAR S.p.A., ALF-X Surgical Robotics Department, Trezzano Rosa, Milan, Italy), which was introduced in 2015 [18]. Telelap Alf-x, by providing a haptic feedback to the surgeon, successfully reduced the average time of experimental cholecystectomy by 60 minutes [12,17-18]. This system was also equipped with an eye-tracking technology, which halts the movements of the robotic arms when the operator’s eyes are not fixed on the screen [12,17-18] and provided an added cost-effectiveness by using low-cost reusable parts [12,18]. In 2017, Su et al. presented a new MRI-guided telesurgery system that performs percutaneous interventions and provides haptic feedback to the operator using varying degrees of pneumatic pressure [19].

Emerging telesurgical technologies 

Although in its infancy, tele-neurosurgical technology is currently being explored. In 2007, O’Malley and Weinstein conducted a successful cadaveric skull base surgery using a trans-oral approach [6,20]. And about a decade later, Wirz et al. presented an endonasal feasibility study for trans-sphenoidal resection of a pituitary tumor with 10 milliseconds of latency time, with the surgeons agreeing that using the device did not feel significantly different from handling a conventional endoscope [16].

Zhao et al. recently published a feasibility study of the integration of a floating 3D visual feedback system in telesurgery [9]. This integration allows multiple surgeons to see a floating, holographic image of the surgical field simultaneously, enhancing the detail of the shared visual display and real-time collaborations amongst medical professionals across the border [9].

Current limitations of telesurgery's clinical translation

Despite the introduction of numerous telesurgical innovations since 2001, only a single randomized controlled telesurgery trial of percutaneous access of the kidney with a remote center of motion active robotic device (PAKY-RCM) exists to date [1]. In addition to the need for further optimization of visual display, latency time, and haptic feedback technology, further randomized controlled trials should be performed to lead to the innovations’ successful clinical translation [17-18]. Other main factors that prevent the implementation of telesurgical technology into today’s clinical setting are summarized in Table 2. If such limiting factors are not dealt with prior to launching the clinical use of this technology, malpractice, the inefficiency of care, and unnecessary spending may result [4].

**Table 2:** A summary of factors that currently limit the use of telesurgery in the real world

Factors that limit Telesurgery’s Clinical Translation
Lack of fully developed training programs and standard operating protocols (including that for equipment maintenance)
The difficulty of the acquisition of equipment
Need for development of a global network
Billing issue on distributing operation fee and facility fee among the participating medical centers
Funding issues
Legal issues, which vary across state and country borders

## Conclusions

The use of telesurgery technology is at a halt and has been since the first and only telesurgery was conducted in 2001. However, with further optimization of visual display, latency time, and haptic feedback technology, design and publication of further randomized controlled trials, and minimization of the factors that limit its clinical translation, telesurgery’s widespread implementation in clinical settings will become highly feasible and geographical barriers will be eliminated.

## References

[1] Raison N, Khan MS, Challacombe B (2015). Telemedicine in surgery: what are the opportunities and hurdles to realising the potential?. Curr Urol Rep.

[2] Hougen HY, Lobo JM, Corey T (2016). Optimizing and validating the technical infrastructure of a novel tele-cystoscopy system. J Telemed Telecare.

[3] Cazac C, Radu G (2014). Telesurgery - an efficient interdisciplinary approach used to improve the health care system. J Med Life.

[4] Satcher RL, Bogler O, Hyle L (2014). Telemedicine and telesurgery in cancer care: inaugural conference at MD Anderson Cancer Center. J Surg Oncol.

[5] Shenai MB, Tubbs RS, Guthrie BL, Cohen-Gadol AA (2014). Virtual interactive presence for real-time, long-distance surgical collaboration during complex microsurgical procedures. J Neurosurg.

[6] Newman JG, Kuppersmith RB, O'Malley BW (2011). Robotics and telesurgery in otolaryngology. Otolaryngol Clin North Am.

[7] Korte C, Nair SS, Nistor V, Low TP, Doarn CR, Schaffner G (2014). Determining the threshold of time-delay for teleoperation accuracy and efficiency in relation to telesurgery. Telemed J E Health.

[8] Xu S, Perez M, Yang K, Perrenot C, Felblinger J, Hubert J (2015). Effect of latency training on surgical performance in simulated robotic telesurgery procedures. Int J Med Robot Robotics Comput Assist Surg.

[9] Zhao D, Ma L, Ma C, Tang J, Liao H (2016). Floating autostereoscopic 3D display with multidimensional images for telesurgical visualization. Int J Comput Assist Radiol Surg.

[10] Shenai MB, Dillavou M, Shum C (2011). Virtual interactive presence and augmented reality (VIPAR) for remote surgical assistance. Oper Neurosurg.

[11] Ghorbanian A, Zareinejad M, Rezaei SM, Sheikhzadeh H, Baghestan K (2013). A novel control architecture for physiological tremor compensation in teleoperated systems. Int J Med Robotics Comput Assist Surg.

[12] Stark M, Benhidjeb T, Gidaro S, Morales ER (2012). The future of telesurgery: a universal system with haptic sensation. J Turk Ger Gynecol Assoc.

[13] Veluvolu KC, Ang WT (2011). Estimation of physiological tremor from accelerometers for real-time applications. Sensors (Basel).

[14] Nguan C, Miller B, Patel R, Luke PP, Schlachta CM (2008). Pre-clinical remote telesurgery trial of a da Vinci telesurgery prototype. Int J Med Robotics Comput Assist Surg.

[15] Xu S, Perez M, Yang K, Perrenot C, Felblinger J, Hubert J (2014). Determination of the latency effects on surgical performance and the acceptable latency levels in telesurgery using the dV-Trainer((R)) simulator. Surg Endosc.

[16] Wirz R, Torres LG, Swaney PJ (2015). An experimental feasibility study on robotic endonasal telesurgery. Neurosurgery.

[17] Stark M, Morales ER, Gidaro S (2012). Telesurgery is promising but still need proof through prospective comparative studies. J Gynecol Oncol.

[18] Stark M, Pomati S, D'Ambrosio A, Giraudi F, Gidaro S (2015). A new telesurgical platform--preliminary clinical results. Minim Invasive Ther Allied Technol.

[19] Su H, Shang W, Li G, Patel N, Fischer GS (2017). An MRI-guided telesurgery system using a Fabry-Perot interferometry force sensor and a pneumatic haptic device. Ann Biomed Eng.

[20] O'Malley BW, Weinstein GS (2007). Robotic skull base surgery: preclinical investigations to human clinical application. Arch Otolaryngol Head Neck Surg.

